# Direct BMP signaling to chordoblasts is required for the initiation of segmented notochord sheath mineralization in zebrafish vertebral column development

**DOI:** 10.3389/fendo.2023.1107339

**Published:** 2023-05-08

**Authors:** Hans-Martin Pogoda, Iris Riedl-Quinkertz, Matthias Hammerschmidt

**Affiliations:** ^1^ Institute of Zoology – Developmental Biology, University of Cologne, Cologne, Germany; ^2^ Cluster of Excellence, Cellular Stress Responses in Aging-Associated Diseases (CECAD) Cluster of Excellence, University of Cologne, Cologne, Germany; ^3^ Center for Molecular Medicine Cologne (CMMC), University of Cologne, Cologne, Germany

**Keywords:** notochord, chordoblast, BMP, retinoic acid, vertebral body, centra, zebrafish, vertebral column

## Abstract

The vertebral column, with the centra as its iteratively arranged building blocks, represents the anatomical key feature of the vertebrate phylum. In contrast to amniotes, where vertebrae are formed from chondrocytes and osteoblasts deriving from the segmentally organized neural crest or paraxial sclerotome, teleost vertebral column development is initiated by chordoblasts of the primarily unsegmented axial notochord, while sclerotomal cells only contribute to later steps of vertebrae formation. Yet, for both mammalian and teleostean model systems, unrestricted signaling by Bone Morphogenetic Proteins (BMPs) or retinoic acid (RA) has been reported to cause fusions of vertebral elements, while the interplay of the two signaling processes and their exact cellular targets remain largely unknown. Here, we address this interplay in zebrafish, identifying BMPs as potent and indispensable factors that, as formerly shown for RA, directly signal to notochord epithelial cells/chordoblasts to promote *entpd5a* expression and thereby metameric notochord sheath mineralization. In contrast to RA, however, which promotes sheath mineralization at the expense of further collagen secretion and sheath formation, BMP defines an earlier transitory stage of chordoblasts, characterized by sustained matrix production/*col2a1* expression and concomitant matrix mineralization/*entpd5a* expression. BMP-RA epistasis analyses further indicate that RA can only affect chordoblasts and their further progression to merely mineralizing cells after they have received BMP signals to enter the transitory *col2a1*/*entpd5a* double-positive stage. This way, both signals ensure consecutively for proper mineralization of the notochord sheath within segmented sections along its anteroposterior axis. Our work sheds further light onto the molecular mechanisms that orchestrate early steps of vertebral column segmentation in teleosts. Similarities and differences to BMP’s working mechanisms during mammalian vertebral column formation and the pathomechanisms underlying human bone diseases such as Fibrodysplasia Ossificans Progressiva (FOP) caused by constitutively active BMP signaling are discussed.

## Introduction

Initially discovered as components of decalcified bone that are capable of inducing ectopic bio-mineralization upon *in vivo* administration into skeletal muscle (1, 2), Bone Morphogenetic Proteins (BMPs) are known to play multiple and diverse crucial roles in different steps of bone formation and homeostasis (3, 4). In the context of vertebral fusions in mice and humans, different effects of BMP signaling have been reported: Recessively inherited loss-of-function mutations in BMP13, also named GDF6 (5), or its close relative GDF3 (6) cause Klippel-Feil-Syndrome (KFS), characterized by abnormal fusion of any two of the first seven cervical vertebrae in the neck, but usually restricted to vertebrae C1 – C3, all of which result from aberrant segmentation along the embryo’s developing axis during the first 3 to 8 weeks of gestation (7). In contrast, dominantly inherited gain-of-function mutations in the BMP receptor ACVR1/ALK2, which render the receptor constitutively active even in the absence of BMPs (8) and that are further activatable by Activin, a member of the other TGFβ superfamily subgroup (9), cause Fibrodysplasia Ossificans Progressiva (FOP) (9–11). FOP is characterized by multiple orthotopic (skeletal) and heterotopic ossifications, including orthotopic fusions at the level of lateral masses and spinous processes of cervical vertebrae, but, in contrast to KFS, not at the level of vertebral bodies themselves. Furthermore, in contrast to the rather early onset of cervical KFS defects, such orthotopic fusions in FOP are only constituted shortly after birth, but progress as the patients age (12).

Multiple and central roles during skeletogenesis have also been assigned to retinoic acid (RA), a derivative of vitamin A (13–15), which cooperates with BMPs to accelerate different processes of cartilage and bone formation and bone turnover *in vivo* (14, 16). To our knowledge, no mutations in genes encoding components or regulators of RA signaling have been assigned to human congenital vertebral malformations as yet, including wedge or hemivertebra as well as vertebral segmentation defects of any kind, most likely due to early lethality caused by such mutations in affected individuals (17). However, in mouse, gain of endogenous RA signaling caused by pharmacological inhibition of the RA-metabolizing enzyme Cyp26 has been shown to cause – among other defects (18) - similar FOP-like fusions of cervical vertebrae (19) as obtained upon gain-of-function of endogenous BMP signaling caused by loss-of-function mutations in the secreted BMP inhibitor Noggin (12, 20), pointing to a possible concerted action of RA and BMP signaling in promoting vertebral fusions. However, negative interplays between BMP and RA signaling have been described as well, including an inhibitory effect of RA- on BMP- signaling in the context of FOP (10) (see also Discussion).

Here, we study the cooperation of BMP and RA signaling during early development of vertebral centra anlagen in the zebrafish. Although initial steps of vertebra formation in teleosts are not driven by scleroblasts from the segmentally organized paraxial mesoderm *via* endochondral ossification as in amniotes, but instead by cells of a primarily unsegmented structure, the notochord (21–25), *via* the mineralization of a non-cartilaginous, type II collagen-rich extracellular matrix (ECM), gain of both RA or BMP signaling at early larval stages lead to an aberrant fusion of developing vertebral centra anlagen in zebrafish (19), somehow comparable to their aforementioned effects in mouse (12, 19, 20).

During larval stages, the notochord of teleosts consists of centrally positioned chordocytes and an outer epithelial layer of chordoblasts. Chordocytes are localized in inner regions of the notochord and are comparatively large cells with characteristic liquid-filled vacuoles. They align as horizontal stacks to provide the hydrostatic core part of the axial strut. The pile of such voluminous chordocytes gets encircled by a layer of rather flat, tightly linked chordoblasts. Thereby, the latter constitute the outer, epithelial border of the cellular notochord (26). Noteworthy, both notochordal cell-types are ontogenetically related, since chordoblasts segregate from early vacuolated notochord cells at the end of segmentation stages (24, 27). Aside from simply configuring the notochord epithelium, chordoblasts exhibit remarkable osteogenic properties during early vertebral column development in teleosts: like bona fide osteoblasts they unidirectionally secrete a collagenous matrix until it evenly envelopes the entire notochord, thereby constituting the so-called notochord sheath. The core part of the sheath consists of a collagen type II-based ECM. According to our own histological analyses (24, 27) the corresponding layers of collagen fibers encircle the notochord of early zebrafish larvae in an orientation perpendicular to the rostro-caudal axis of the organ. This central collagenous layer of the sheath (*elastica media*) itself is enveloped by a thin (inner) and thicker (outer) elastin-based basal lamina, the *elastica interna* and *elastica externa*, respectively. The chemical composition of the notochord sheath thus is very similar to that of cartilage matrix and designed to withstand the hydrostatic pressure generated by the vacuolated chordocytes (28). Importantly, the notochord sheath can also serve as a substrate for mineralization (which accordingly can be compared with cartilage mineralization during chondral ossification), with iterative sections of it calcifying to form the chordacentra, repetitive mineralized ring-shaped fractions of the *elastica media* that serve as the precursor elements of the eventual vertebrae. Later, outer autocentra and vertebral arches are added to these chordacenta, mainly driven by sclerotomal cells derived from the paraxial somites (26, 28–30).

Of note, genetic ablation of zebrafish chordoblasts at a time point when a proper notochord sheath has already been formed, abrogates subsequent chordacentra mineralization (24). This demonstrates that chordoblasts do not solely produce notochord-derived ECM but later also actively fuel its mineralization. For this goal, segmentally arranged, ring-shaped clusters of chordoblasts start to express Ectonucleoside Triphosphate Diphosphohydrolase 5a (Entpd5a), an enzyme that is indispensable for ossification (31) and that provides inorganic mono-phosphate for matrix mineralization (23–25). Once metameric notochord sheath calcification has been initiated and dedicated chordacentra start to grow in length and thickness, the underlying chordoblasts progressively attenuate expression of collagens, while matrix proteins remain to be produced and secreted in intervertebral regions (24, 25). In this context, Wopat and colleagues were able to distinguish three subpopulations of chordoblasts: merely *collagen* genes-expressing, transitory *collagen*- and *entpd5a*-coexpressing, and *entpd5a*-positive, but *collagen*-negative cells (25). These findings point to an at least two-step developmental progression of chordoblasts from matrix-producing to matrix-mineralizing cells. A progression from matrix-producing to matrix-mineralizing properties has also been described for the transitioning of osteoblasts to pre-osteocytes (32, 33). During calvarial plate formation *via* intramembranous ossification in zebrafish, this transition has been shown to be driven by RA signaling (34), and a corresponding effect of direct RA signaling to chordoblasts has been shown to drive their transformation to mineralizing skeletogenenic cells during zebrafish backbone development (24). However, it remained unclear where exactly in this two-step process RA acts and how it interacts with BMP, for which a similar mineralization-promoting effect during zebrafish vertebral column formation had been formerly reported (19).

Performing detailed comparative analyses of zebrafish vertebral column defects caused by gain- or loss of BMP- or RA-signaling, we demonstrate that direct BMP signaling to chordoblasts promotes their transition from purely matrix-generating cells to the transitory cell type that simultaneously generates and mineralizes ECM. In contrast, RA promotes the progression of this transitory cell type to purely ECM-mineralizing cells. Epistasis analyses combining loss of BMP signaling with gain of RA signaling further indicate that RA can only do so after chordoblasts have entered the BMP-induced transitory stage. However, this consecutive action does not involve a stimulation of RA activity by BMP signaling.

In light of these findings, we discuss that, rather than RA (19, 22, 24, 35), it might be BMP signaling that qualifies as the initial pattern generator regulating the segmentation of the notochord sheath and thus the formation of iterative vertebral body anlagen from the foremost unsegmented chordoblast epithelium and notochord sheath. Similarities and differences between the roles of BMPs during vertebral column formation and segmentation in zebrafish versus mammals are discussed.

## Materials and methods

### Zebrafish lines

The following zebrafish lines were used: *Tg(R2-col2a1a:EGFP)^nu13Tg^* to visualize *col2a1* expressing chordoblasts (27), *Tg(cyp26b1:YFP)^hu7426^* to visualize RA-responsive chordoblasts (24), *Tg(col2a1a:CFP-NTR)^fr42Tg^* to ablate *col2a1a* expressing chordoblasts (24), *Tg(hsp70:bmp2b)^fr13Tg^* (36) and *Tg(hsp70:noggin3)^fr14Tg^* (36) to globally induce *bmp2b* expression or to globally inhibit all endogenous BMP signaling upon heat-shocking (temporal control), respectively. The line *Tg(entpd5a:EGFP)^fr53Tg^* was generated in the course of this study (details see below) with the goal to label chordoblasts that exhibit mineralizing activity. Larvae were taken for *in vivo* imaging or fixed for subsequent histology at the ages of 5, 6, 7 or 8 days post fertilization (dpf) with notochord lengths ranging from 3.2 to 3.6 mm, respectively. Ages of the specimens are specified in the figures. All zebrafish experiments were approved by the local and federal animal care committees in Cologne (LANUV Nordrhein-Westfalen: 84-02.04.2012.A251, 84-02.04.2012.A390, 81-02.04.40.2022.VG005, 81-02.04.2018.A282, 81-02.04.2022.A104; City of Cologne: 8.87-50.10.31.08.129).

### RA, BMS493, Metronidazole and heat-shock treatments

A 10 mM stock solution of *all*-*trans* RA (Sigma; R2625) in DMSO as well as a corresponding working dilution was prepared as described (37). In order to globally activate RA signaling, wild-type or transgenic zebrafish larvae were incubated with final concentrations of 1 μM RA from 4 dpf until phenotypic evaluation or RNA extraction at 7 dpf or 8 dpf. For the reverse approach (global blockage of RA signaling) the pan-RAR antagonist BMS493 (Sigma; B6688) (38) using a 10 mM stock solution in DMSO was prepared. Transgenic larvae were incubated with a final concentration of 7 µM BMS493 from 4-6 dpf. As controls, siblings were treated with equivalent concentrations of ethanol (1 °/_oo_)_/_DMSO (0.1 °/_oo_) (RA) or DMSO (0.7 °/_oo_) alone (BMS493).

To show that chordoblasts are essential for BMP regulated notochord sheath mineralization it requires (a) the absence of the chordoblast layer while (b) simultaneously having a rather complete notochord sheath secreted in (c) otherwise intact larvae (d) at proper developmental stages. These conditions cannot be provided by any mutant or morphant background known to date. Thus, the approach of temporally-controlled chordoblast ablation was inevitable to answer that question. Treating Tg(*R2-col2a1a:cfp-nfsb*) larvae with Metronidazole (Mtz) as described below does not result in notable developmental retardation and affected tissues outside the notochord [e.g. paraxial mesoderm; see **Figure S1** and (24)]. For genetic cell ablation *via* the Nitroreductase-Metronidazole (Mtz) system, Mtz (Sigma-Aldrich; M3761) (39) was freshly dissolved in E2 embryo medium (15 mM NaCl, 0.5 mM KCl, 0.1 mM MgSO_4_, 150 µM KH_2_PO_4_, 50 µM Na_2_HPO_4_) and replaced every day of treatment. For chordoblast ablation larvae were treated with 7 mM Mtz from 4-5 dpf and with 5 mM Mtz for the following 1.5 days. All treatments were performed in the dark in a 28°C incubator. For heat-shock experiments to globally induce the expression of the corresponding effector-transgenes Tg(*hsp70:bmp2b*) and Tg(*hsp70:noggin3*), the embryo medium was rapidly replaced by 40°C prewarmed medium. Subsequently, the larvae were left for 45 min in a 40°C incubator and then transferred back to 28°C for slow cool-down to standard conditions. In case of co-treatments with drugs, the reagents were left out of the embryo medium while heat-shocking.

### Plasmid construction, injections and transgenesis

The *Tg*(*entpd5a:egfp)^fr53Tg^* transgene was generated following standard procedures (40) using the BAC CH211-202H12 construct that had previously been reported to serve as a read-out for *entpd5a* expression (31). Plasmids for chordoblast-specific (de)activation of BMP signaling *(R2-col2a1a:dn-bmpr1b-p2a-dTomatoCAAX* and *R2-col2a1a:ca-bmpr1b-p2a-dTomatoCAAX)* were cloned using Multistep Gateway Recombineering system (Invitrogen) and the Tol2kit (41, 42). For the construction of dedicated middle-entry clones, the p2a-dTomatoCAAX sequence (Addgene) was first PCR amplified and hereby equipped with an *Xba*I site 5’-wards and a *Not*I-site at the 3’-side. Thereafter, the obtained fragment was cloned into pME-MCS (42) using *Xba*I and *Not*I restriction sites. The resulting intermediate-construct was digested with *Eco*RI/*Xba*I to be used as receiving vector in a ligation with *dn-bmpr1b* ORF from *Xenopus* as insert that was obtained *via EcoR*I/*Xba*I digest from a *hsp70:dn-bmpr1b-egfp* construct (43).

In preparation for the other middle-entry clone, the ORF of mouse *ca-bmpr1b*, which has been formerly shown to function properly also in zebrafish, was PCR-amplified from the *HSE:cBmpr1b, EGFP* construct (44), adding a *Spe*I site at its 5’ and a *Xba*I site at its 3’ end. Subsequently, the fragment was cloned in frame *via* these cut sites into the aforementioned and accordingly prepared pME-MCS-p2a-dTomatoCAAX vector.

**Figure 1:**
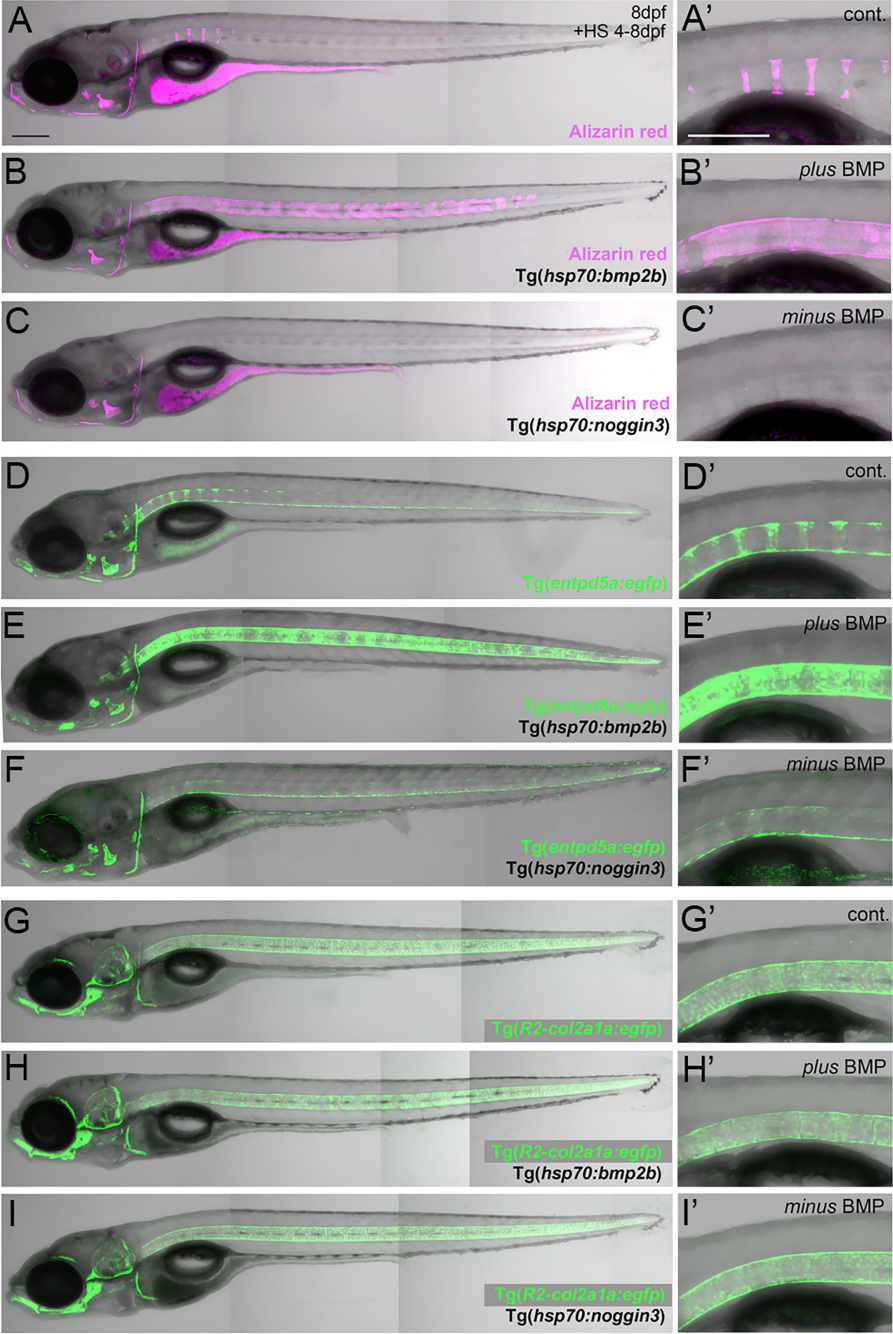
BMP regulates mineralization and *entpd5a* expression along the notochord. **(A–I)** Lateral views of zebrafish larvae shown in merged transmitted and fluorescent light channels with tissue staining and genotypes as indicated at an age of 8dpf. Alizarin red labels mineralized extracellular matrix (ECM) in magenta **(A–C)**. *entpd5a*-expressing cells are visualized by Tg(*entpd5a:egfp*) in green **(D–F)**, *col2a1*-expressing chondroblasts and chordoblasts by Tg(*R2-col2a1a:egfp*) in green **(G–I)**. Magnified views of the region right dorsal to the swim bladder from corresponding larvae are shown in **A**’ o **I**’. All larvae have been heat-shocked once a day for 45 minutes at 40°C from 4-8 dpf. Note the ventral line of chordoblasts along the entire length of the notochord that remained EGFP positive after *noggin3* overexpression **(F, F’)**, pointing to BMP-independent regulation in this particular notochordal subdomain. Scale bars: 200 µm.

The final *R2-col2a1a:ca-/dn-bmpr1b-p2a-dTomatoCAAX* constructs were generated *via* LR recombination of either of the two constructed middle-entry vectors together with p3E-polyA, pDestTol2CG and p5E-*R2-col2a1a* (24). All primers used for cloning procedures are listed below. Mosaic G0 larvae (**Figures 3P–R**) and the stable *entpd5a:egfp* transgenic line (**Figures 1**, **3**, **4**) were generated by standard injection and visual screening (fluorescence microscopy) procedures. Injection solutions contained nucleic acids (plasmid DNA, BAC DNA, Tol2mRNA respectively) at a concentration of 50 ng/µl each in 5% Phenol red (Sigma; P0290) and 1x Danieau buffer (17.4 mM NaCl, 0.21 mM KCl, 0.12 mM MgSO4, 0.18 mM Ca(NO3)_2_, 1.5 mM HEPES, pH 7.6). Tg(*entpd5a:egfp*)-positive carriers were subsequently crossed to homozygous Tg(*hsp70:bmp2b*) and Tg(*hsp70:noggin3*) fish to generate the respective double-transgenic fish with which the experiments were conducted.

### Tissue cryo-sectioning and immunofluorescence stainings

For cryo-sectioning samples fixed in 4% PFA (Sigma; P6148) and stored in 100% MeOH at -20°C were progressively rehydrated to PBST (PBS/0.1% Tween-20), embedded in a solution of PBS containing 1.5% low-melting agarose and 15% sucrose, and incubated overnight at 4°C in 30% sucrose in PBS. Tissue blocks covered in cryomatrix (VWR, Radnor, PA) were snap frozen in 2-methylbutane (Isopentane; Carl Roth, Karlsruhe, D) at -80°C. Sagittal as well as cross sections of 14 µm thickness were prepared from the specimen’s anterior half of the abdominal domain using a Leica CM 1850 cryostat, collected on coated glass slides (Ultra Plus; VWR, Radnor, PA) and stored at –20°C until further processing. At least one hour prior to usage, frozen cryostat sections were thawed at room temperature. For subsequent immunofluorescence studies, sections were equilibrated by three washes of five minutes each in PBTX (PBS, 0.3% Triton-X) followed by at least two hours blocking in block solution (10% fetal calf serum, 1% DMSO in PBTX) at room temperature. Stainings were performed with the primary antibodies chicken anti-GFP (ThermoFisher Scientific, A-10262) and rabbit anti-phosphoSMad1/5/8 (Cell Signaling/NewEnglandBiolabs, #9511, http://zfin.org/ZDB-ATB-081015-1#summary), and the secondary antibodies goat-anti-chicken AlexaFluor-488 (ThermoFisher Scientific, A-11039) and goat-anti-rabbit AlexaFluor-555 (ThermoFisher Scientific, A-21428). Primary antibodies were diluted 1:500, secondary antibodies 1:1000 in block solution. Incubations with antibodies were carried out at 4°C over night, followed by six washes in PBTX for 15 minutes each. Control stains were performed on consecutive sections while omitting the incubation with the corresponding primary antibody. Prior to imaging, stained sections were mounted with Mowiol/Dapi mixture and left in the dark to dry at room temperature for 24 hours.

### Imaging

Conventional fluorescent light microscopy was performed using a Zeiss AxioImager.M2 in combination with Axiovision 4.0 software. Confocal z-stacks were recorded using a Zeiss LSM 710 microscope. Fluorescent signals were excited by appropriate wavelengths of a 1-photon laser and detected sequentially in separate channels. For *in vivo* confocal imaging, whole larvae were anesthetized with 0.015% Tricaine and mounted in 1.5% low melting agarose/0.015% Tricaine in embryo medium. For subsequent image processing, ZEN *black* (Zeiss) and Photoshop CS4 (Adobe) software was used.

### TEM analysis and alizarin red *in vivo* stains

For transmission electron microscopy (TEM) to assay notochord sheath diameter and integrity, 8 dpf zebrafish larvae with a notochord length of 3.5 mm were fixed in 2.5% glutaraldehyde and 2% PFA in 1x PBS firstly for 30 minutes at room temperature and then at 4°C overnight. Decalcification was not performed, since overall mineralization is little advanced at this early stage. Fixed specimens were then washed several times in 1x PBS and two times in 10% PBS, post-fixed with 1% osmium tetraoxide in 10% PBS for 1 hour at 4°C, washed three times in 10% PBS and incubated in 1% uranylacetate for 30 minutes at 4°C, dehydrated in a graded series of ethanol, and embedded in araldite (Hundsman Advanced Materials, Derry, NH). Ultrathin (70 nm) and consecutive semithin (750nm) cross-sections of the specimen’s abdominal region were cut using the ultramicrotome Leica EM UC7. Ultrathin sections were mounted on pioloform-covered copper grids and post stained with aqueous uranyl acetate (2%, 10 minutes) and Reynolds’ lead citrate (3.5 minutes). The samples were examined with the transmission electron microscope CM 10 (FEI Europe Main Office, Eindhoven, The Netherlands). Micrographs were taken with the ORIUS SC200W TEM CCD camera using the software DigitalMicrograph that was also used for primary micrograph internal measurements (Gatan Inc., Pleasanton, CA). Semithin consecutive sections were collected on regular microscope slides (VWR) and stained with a filtered aqueous solution of 0.5% Azur II, 0.5% Methylene blue and 0.5% Borax for 30 seconds on a 70°C pre-warmed heating plate. After thoroughly rinsing the stained sections with distilled water, they were dried on a heating plate bevor mounting in Moviol and adding a cover slip.

Alizarin red stains on living larvae were performed as described (24). For ages and corresponding notochord lengths of stained larvae please see Materials and methods’ paragraph “Zebrafish lines”. Except for **Figure 1** showing total larvae, the phenotypic evaluation of notochord sheath mineralization *via* Alizarin red stains was conducted at the prospective Weberian and abdominal domains.

### qPCR

**Figure 2:**
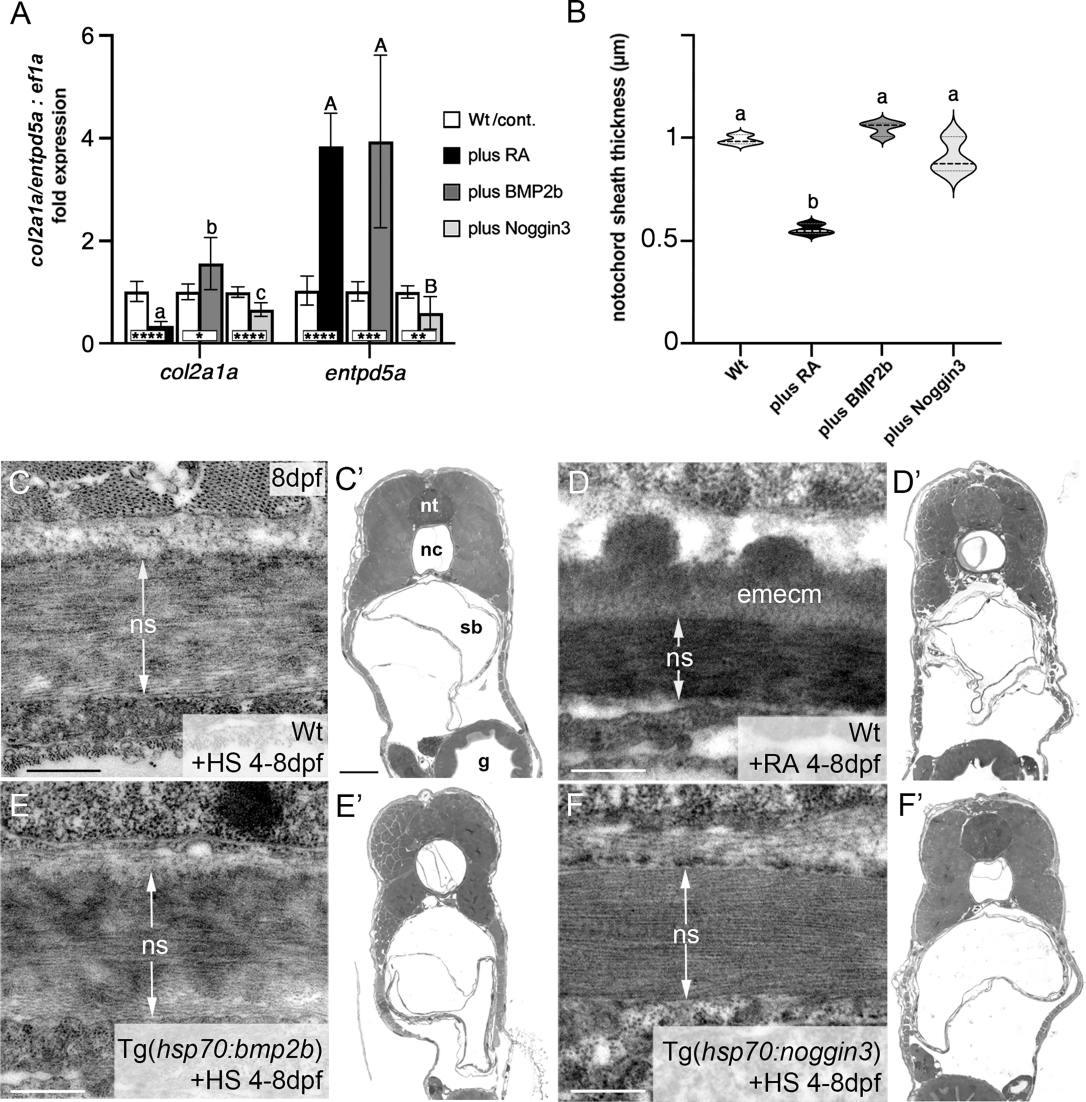
BMP has no major impact on *col2a1* expression and notochord sheath formation. **(A)** qPCR results for the matrix-generating gene *col2a1a* and the matrix-mineralizing gene *entpd5a* after RA exposure, BMP2b or Noggin3 overexpression, respectively. See main text for exact experimental conditions. Each shaded column indicates the ratio of expression levels between an experimental group and its specific control group set to a value of 1 (white columns), with the respective statistical significances determined *via* Student’s t-test with Welch’s correction and indicated as * (significant with p<0.05), ** (significant with p<0.01), *** (significant with p<0.001) and **** (significant with p<0.0001). In addition, the letter subscript system (small letters for *col2a1a*; capital letters for *entpd5a*) is used to indicate significances of differences between the different experimental groups, with groups with the same letters being not significantly different according to Brown-Forsythe and Welch ANOVA tests (p < 0.0001) with *post-hoc* Dunnett’s T3 multiple comparison test (p < 0.05). **(B)** Quantitative analysis of notochord sheath thickness, based on measurements performed on TEM specimens as in **(C–F)**. Listed conditions have been obtained *via* drug treatment (RA) or by the aforementioned heat-shock inducible transgenes; violin plots of N=3 biological samples per condition from independent spawnings/treatments, with each N representing the mean value from 5 different sections per specimen; broken and dotted lines indicate mean and quartiles, respectively; experimental groups labeled with identical superscript letters are not significantly different according to Student’s t-test with Welch’s correction, p < 0.05. **(C–F)** TEM micrographs of cross-sections through the abdominal notochord sheath from larvae with genotypes and treatments as indicated; differences in the darkness of the notochord sheath are due to differences in its mineralization levels in the different conditions. **(C’–F’)** show overviews of the corresponding semithin-sections indicating that the TEM images were taken at corresponding positions along the antero-posterior axis of the specimens. Scale bars: 0.5 µm **(C–F)**, 50 µm **(C’)**; g, gut; nc, notochord; ns, notochordal sheath; nt, neural tube; emecm, external mineralized extracellular matrix; sb, swim bladder.

Per condition (**Figure 2A**), total RNA was isolated from trunks and tails of 3x30 larvae (N=3) of an age of 8 dpf that had been decapitated posterior of the inner ear, using Trizol Reagent (Thermo Fisher Scientific) with the PureLink RNA Mini Kit (Thermo Fisher Scientific), including on-column DNaseI. Quality and amount of the extracted RNA was determined *via* a Quantus**™** fluorometer (Promega) by assaying 260/280nm and 260/230nm ratios. Gene expression was assayed using the Luna RT-qPCR Kit (New England Biolabs, E3006L) in combination with TaqMan assays (*col2a1a* (NCBI accession number XM_005166863.4: premade assay Dr03099270_m1; *entpd5a* (NCBI accession number XM_679770.8: custom made assay, forward primer AGCCCGGACTGTCAGCATAT, reverse primer TCAACAGCTGCACAATGGATTC, probe primer TATGCCTGAAAAGGGTGG on an ABI-PRISM 7500 Fast Detection system. As endogenous internal controls the housekeeping genes *ef1a* (assay ID Dr03432748_m1; NCBI accession number AM422110.2), *rps23* (assay ID Dr03430371_m1; NCBI accession number NM_001110121) and *ppiaa* (assay ID Dr03152038_m1; NCBI accession number NM_212758) have first been evaluated for their suitability in the context of applied conditions (controls, +RA, +BMP2b, +Noggin3) *via* the Excel-based tool *BestKeeper* (https://www.gene-quantification.de/bestkeeper.html; (45)): SDs of C**_T_** values were 0.12 (*ef1a*), 0.19 (*rps23*) and 0.29 (*ppiaa*), SDs in regards to resulting fold change of expression level were 1.09 (*ef1a*), 1.14 (*rps23*) and 1.22 (*ppiaa*). Thus, all three chosen reference genes are within the range of stable expression and do not show critical changes in their activities for analyzed conditions. This was further confirmed *via* a gene stability test applying the *Genorm* algorithm (http://blooge.cn/RefFinder/?type=reference), which revealed same stability for *ef1a* and *rps23* (both 0.416), while the value for *ppiaa* was moderately inferior (0.435), with values closer to 0 representing higher stability. Based on this and the lower variances in C**_T_** values we decided to use *ef1a* as internal reference gene for normalization.

For the final qPCR experiments, each condition was assayed in biological triplicates (N=3) and with technical triplicates (n=3) per biological sample. PCR conditions were set up according to the instructions given by the Luna RT-qPCR Kit that includes the cDNA synthesis step: reverse transcription for 10 minutes at 55°C, initial denaturation for 1 minute at 95°C, 42 cycles with 1 minute denaturation at 95°C and extension for 30 seconds at 60°C. Relative differences were calculated using the ΔΔC_T_ method *via* the 7500 Software version 2.3 (ABI). Subsequent statistical analysis of biological samples and experimental groups was conducted with Prism8.4.2 software (GraphPad Software).

### Statistics

Statistical analyses were performed using Prism8.4.2 software (GraphPad Software). Data points of qPCR samples (**Figure 2A**) passed the D’Agostino and Pearson omnibus normality test and thus are not inconsistent with a normal distribution. Since subsequent F tests revealed significantly different variances among samples, Student t-test with Welch’s correction was used for comparisons between each experimental group and its corresponding control group (*,**,***,****: statistically significant with p < 0.05, p<0.01, p<0.001, p<0.0001, respectively). Furthermore, statistical significance among the three experimental groups (+RA, +BMP2b, +Noggin3) per analyzed marker gene was determined by parametric Brown-Forsythe and Welch ANOVA tests with *post-hoc* Dunnett’s T3 multiple comparison test (significance threshold of p < 0.05).

Notochord sheath thickness (**Figure 2B**) was measured using Fiji software. The mean values of five measured data points per biological sample were used for final statistical analysis, with N=3 biological samples per condition. Here, no inconsistence with a normal distribution was determined by the Shapiro-Wilk test. Testing for significance between shown groups was performed *via* Student t-tests with Welch’s correction (significance threshold of p < 0.05).

### Primers

entpd5a-egfp-F ACCACCAGTCAAGCCTTCAGCTGTTTGCAGTGTCGGCGTGGGAGAAAGAAACC**atggtgagcaagggcgaggag**

entpd5a-Kan-R CCGGCAAAAAGCCACATGGACACCAACGTCAAGTGCAACATCTGCTGAGA**ggactagtctattccagaagtagtgaggag**

XbaI-P2A-FTTAGTCTAGAGGCTCCGGAGCCACGAACTT

NotI-caax-RTTAGGCGGCCGCTCAGGAGAGCACACACTTGC

SpeI-mAlk6CA-FTTAGACTAGTCCACCATGCTCTTACGAAGCTCTGG

XbaI-mAlk6CA-RTTAGTCTAGAGAGGCTAGCATAATCAGGAA

## Results

### Like RA, BMP signaling is required for notochord sheath mineralization

Zebrafish chordacentrum formation relies on segmented mineralization of the notochord sheath driven by chordoblasts (23–25). It starts anteriorly at the interface of the postcranial and cervical domains of the notochord. From there it progresses sequentially towards the posterior tip of the organ. Proper formation of chordacentra has been formerly shown to require retinoic acid (RA) signaling to chordoblasts, whereas gain of RA signaling leads to premature and ectopic notochord sheath mineralization and the fusion of chordacentra (19, 24, 35). To test whether BMP signaling has a similar impact on segmented notochord sheath mineralization, we made use of heat shock-inducible effector transgenes that allow for temporally controlled global hyperactivation or blockage of BMP signaling, respectively (36). In line with previous findings following an unbiased candidate approach (19), heat-shock induction of the *hsp70:bmp2b* transgene from 4 to 8 days post fertilization (dpf) robustly caused chordacentra fusion along the notochord (N=254/254) (**Figures 1A–B’**). In contrast, corresponding treatment of Tg(*hsp70:noggin3*) larvae to abrogate all endogenous BMP signaling caused the complete absence of sheath mineralization (N=163/163) (**Figures 1C, C’**). Thus, BMP is not only a potent but also an indispensable inducer of axial mineralization at this early phase of chordacentra development.

Ectonucleoside Triphosphate Diphosphohydrolase 5a (Entpd5a) has been formerly shown to be required for bone matrix ossification and notochord sheath mineralization in zebrafish (31). To investigate whether the effect of BMP signaling on notochord sheath mineralization might be due to a corresponding regulation of *entpd5a* expression in chordoblasts, we generated a dedicated EGFP-based reporter transgene *via* BAC recombination following formerly described instructions (31) and established a transgenic *entpd5a:egfp^fr53Tg^* line that recapitulates the recently reported activity of related transgenes (**Figures 1D, D’**) (23, 25). In double transgenics, heat-shock activation of Tg(*hsp70:bmp2b*) from 4–8 dpf led to a strongly up-regulated and uniform, rather than segmented, expression of the *entpd5a:egfp* transgene along the entire notochord (N=189/189) (**Figures 1E, E’**). In contrast, corresponding activation of the *hsp70:noggin3* transgene led to strong reduction of segmented *Tg(entpd5a:egfp)* expression (N=273/273) (**Figures 1F, F’**). Together, this indicates that BMP signaling promotes *entdp5a* expression in chordoblasts and thereby notochord sheath mineralization, similarly to the formerly reported effect of RA (19, 24, 35).

### Unlike RA, BMP signaling does not compromise notochord sheath production

We have formerly shown that upon treatment with RA, enhanced and premature mineralization of the notochord sheath by chordoblasts coincides with an attenuation of their matrix production, reflected by the loss of *col2a1* expression and a reduction in the thickness of the notochord sheath (24). To learn whether this also applies to the effect of BMPs on notochord sheath mineralization, we investigated *R2-col2a1a:egfp* transgene (27) expression under circumstances as described above for Tg(*entpd5a:egfp*). However, neither *R2-col2a1a:egfp*; *hsp70:bmp2b* nor *R2-col2a1a:egfp*; *hsp70:noggin3* double-transgenics displayed remarkable differences in axial Tg(*R2-col2a1a:egfp*) expression levels as detectable *via in vivo* imaging after heat-shock treatments from 4-8 dpf (N=46/46; N=62/62) (**Figures 1G–I’**).

To validate these results, we performed qPCR studies of *entpd5a* and *col2a1a* transcripts with total RNA samples extracted from larvae treated with RA or after transgenic BMP overexpression, respectively (**Figure 2A**). For *entpd5a*, this approach confirmed the data obtained from the *in vivo* imaging of Tg(*entpd5a:egfp*) (**Figures 1D–F**), with gain of both RA or BMP signaling leading to an approximately 4fold increase in *entpd5a* expression (**Figure 2A**). For *col2a1a*, however, only RA treatment led to an approximately 3fold reduction of expression levels, whereas gain of BMP signaling even led to a subtle, but significant increase. Furthermore, loss of BMP signaling upon transgenic overexpression of Noggin3 resulted in moderately, but significantly reduced expression levels of both *col2a1a* and *entpd5a*. Corresponding differences between the effects of BMP and RA were also observed *via* our transmission electron microscopy (TEM) studies of the thickness of the notochord sheath (which mainly contains type II collagen encoded by *col2a1* from chordoblasts). Upon RA treatment, notochord sheath thickness was reduced almost by half, whereas no significant changes were observed by gain- or loss of BMP signaling (**Figures 2B–F**).

Taken together, we conclude that BMP signaling is a positive and indispensable regulator of early chordoblast-specific *entpd5a* expression and thereby of notochord sheath mineralization. However, and in difference to the properties of RA in the same context, it does not tune down *col2a1* expression and extracellular matrix (ECM) production in parallel. In light of the formerly described 2-step progression from ECM-generating to ECM-mineralizing chordoblasts (25) (see Introduction), we hypothesize that BMP signaling accounts for the first step, the transition to the transitory stage with both ECM-generating and ECM-mineralizing properties.

### Chordoblasts are direct targets of BMP signaling

**Figure 3:**
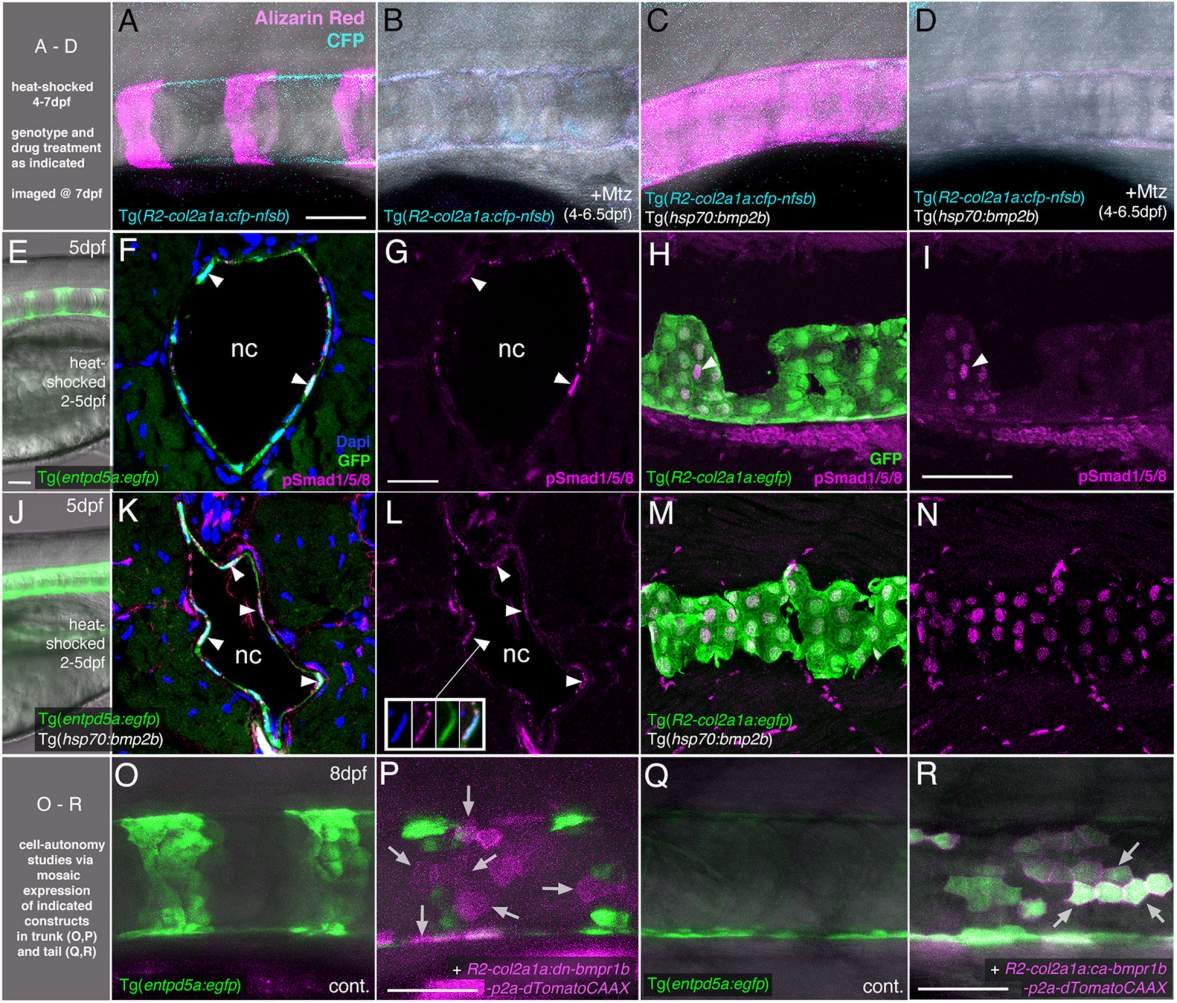
BMP directly targets chordoblasts to regulate Entp5a-mediated mineralization of the notochord sheath. **(A–D)** BMP induced hyper-mineralization of the notochord sheath requires an intact chordoblast layer. **(E–N)** Chordoblasts receive BMP signals as indicated by the presence of the responsible intracellular mediator pSmad1/5/8. Apart from chordoblasts, global overexpression of *bmp2b via* induction of Tg(*hsp70:bmp2b*) also resulted in the gain of pSmad1/5/8-positive cells within the myosepta, muscle tissue and the neural tube **(K-N)**. **(O–R)** Mosaic chordoblast-specific blockage or activation of the intracellular BMP signaling cascade alters *entpd5a* expression autonomously in targeted notochord epithelium cells. **(A–R)** Maximum intensity projections of confocal images with merged channels showing notochords and their immediate surroundings in live whole-mounts **(A–E, J, O–R)** and on stained cryosections **(F, H, K, M)**. **(G, I, L, N)** show corresponding single 555nm channels in magenta. **(E, J)** delineate overviews of those areas of heat-shocked larvae from which the sections presented in the corresponding rows were prepared. **(A–P)** have been assayed in the abdominal region at the level of the swim bladder, **(Q)** and **(R)** in the tail. **(A–E, H–J, M–R)** are lateral views with anterior to the left, **(F, G, K, L)** show transverse sections with dorsal upwards. Arrowheads in **(F, G, I, K, L)** point to exemplary chordoblasts with clear nuclear pSmad1/5/8 staining. Inset in **(L)** depicts magnified single channel images of pSmad1/5/8-positive chordoblast indicated in **(L)** by connected arrowhead. Arrows in **(P)** mark magenta-colored *dn-bmp1b-p2a-dTomatoCAAX* expressing cells that are located in a usually *entpd5a*-positive chordoblast cluster (compare with **(O)**), but that do not express the *entpd5a:egfp* transgene when BMP signal reception is blocked (N=5; n=28/39 dTomatoCAAX-positive, but EGFP-negative cells). Arrows in **(R)** point at a prominent group of chordoblasts that are triple positive for EGFP, the constitutively active BMP receptor Bmpr1b and dTomatoCAAX (N=2, 48 dTomatoCaax and EGFP double-positive of 53 dTomatoCAAX expressing chordoblasts). Ages, treatments, genotypes and color-coded markers as indicated. Scale bars: 50 µm **(A, E, I, O, P)**; 25 µm **(G)**. Mtz, Metronidazole; nc, notochord.

To further prove that the BMP-induced hyper-mineralization (**Figure 1B**) is indeed a consequence of altered activity of chordoblasts (and not other osteogenic cells as for instance scleroblasts), we tested whether BMP overexpression can induce the same phenotype in the absence of an intact notochord epithelium. To do so, we generated Tg(*R2-col2a1a:cfp-nfsb*); Tg(*hsp70:bmp2b*) double-transgenic larvae for temporally controlled and cell type-specific ablation of chordoblasts *via* the Metronidazole/Nitoreductase system (39). While leaving the overall integrity of the larvae intact (**Figure S1** and (24)), Mtz treatment of Tg(*R2-col2a1a:cfp-nfsb*) larvae from 4 - 6.5 dpf resulted in the complete failure of notochord sheath mineralization, as assayed by Alizarin red staining at 7 dpf (N=81/81) (**Figures 3A, B**). This phenotype could not be alleviated or even converted into a hyper-mineralization upon concomitant Tg(*hsp70:bmp2b*) expression from 4-7 dpf (N=112/112), although the latter efficiently induced centra fusion of Tg(*R2-col2a1a:cfp-nfsb*) transgenics in the absence of Mtz (N=43/43; **Figures 3C, D**). Thus, BMP2b-induced mineralization of the notochord sheath depends on functional chordoblasts.

This effect of BMP signaling on chordoblasts could be either direct or indirect. To address these possibilities, we first performed immunofluorescence studies for phosphorylated Smad1/5/8 (pSmad1/5/8), the intracellular BMP signal transducer, in heat-shocked wild-type (**Figures 3E–I**) and Tg(*hsp70:bmp2b*) larvae (**Figures 3J–N**) at 5 dpf, when clearly segmented *entpd5a* expression in the chordoblast layer becomes apparent. Double labeling for transgene-encoded *entpd5a:egfp* (**Figures 3F, G, K, L**) or *col2a1:egfp* (**Figures 3H, I, M, N**) expression revealed nuclear pSmad1/5/8 staining in nearly all *entpd5a:egfp*-positive chordoblasts of wild-type (**Figures 3F, G**; N=4/4) and heat-shocked Tg(*hsp70:bmp2b*) larvae (**Figures 3K, L**; N=4/4) and in all *col2a1*-positive chordoblasts of heat-shocked Tg(*hsp70:bmp2b*) larvae (**Figures 3M, N**; N=5/5), whereas in *col2a1*-positive chordoblasts of wild-type larvae, nuclear pSmad1/5/8 was restricted to metamerically organized clusters that most likely correspond to their mineralizing subsets (**Figures 3H, I**; N=3/3).

Secondly, we performed chordoblast-specific cell-automomous loss- and gain-of-function studies, injecting dominant-negative or constitutively active versions of *bmpr1b* under the control of the chordoblast-specific *R2-col2a1a* enhancer element (*R2-col2a1a:dn-bmpr1b-p2A-dTomatoCAAX* or *R2-col2a1a:ca-bmpr1b-p2A-dTomatoCAAX*; (24, 27, 43, 44)) into *Tg(entpd5a:egfp*) embryos. Even though we made use of the integration-facilitating Tol2 system (41, 42), resultant larvae were mosaic for the injected expression constructs. Larvae injected with the *R2-col2a1a:dn-bmpr1b-p2A-dTomatoCAAX* cassette displayed clearly disrupted Tg(*entpd5a:egfp*) expression in usually *entpd5a*-expressing chordoblast clusters (N=5; n=28/39 dTomatoCAAX-positive, but eGFP-negative cells) (**Figures 3O, P**). Vice versa, we were able to ectopically induce Tg(*entpd5a:egfp*) expression in the notochord epithelium after injecting the *R2-col2a1a:ca-bmpr1b-p2A-dTomatoCAAX* construct, with EGFP and dTomato double-positive chordoblasts even in more caudal regions of the notochord that had not developed any endogenous *entpd5a* expression at 8 dpf as yet (N=2, 48/53 double-positive chordoblasts) (**Figures 3Q, R**).

Together, this indicates that chordoblasts directly receive BMP signals, to which they respond by the initiation of *entpd5a* expression and notochord sheath mineralization. However, in contrast to RA signals, which according to our former studies are also directly received by chordoblasts (24), BMP signaling does not induce the termination of *col2a1* expression and notochord sheath formation.

### RA cannot promote notochord sheath mineralization when BMP signaling is blocked during early phases of mineralization initiation

**Figure 4:**
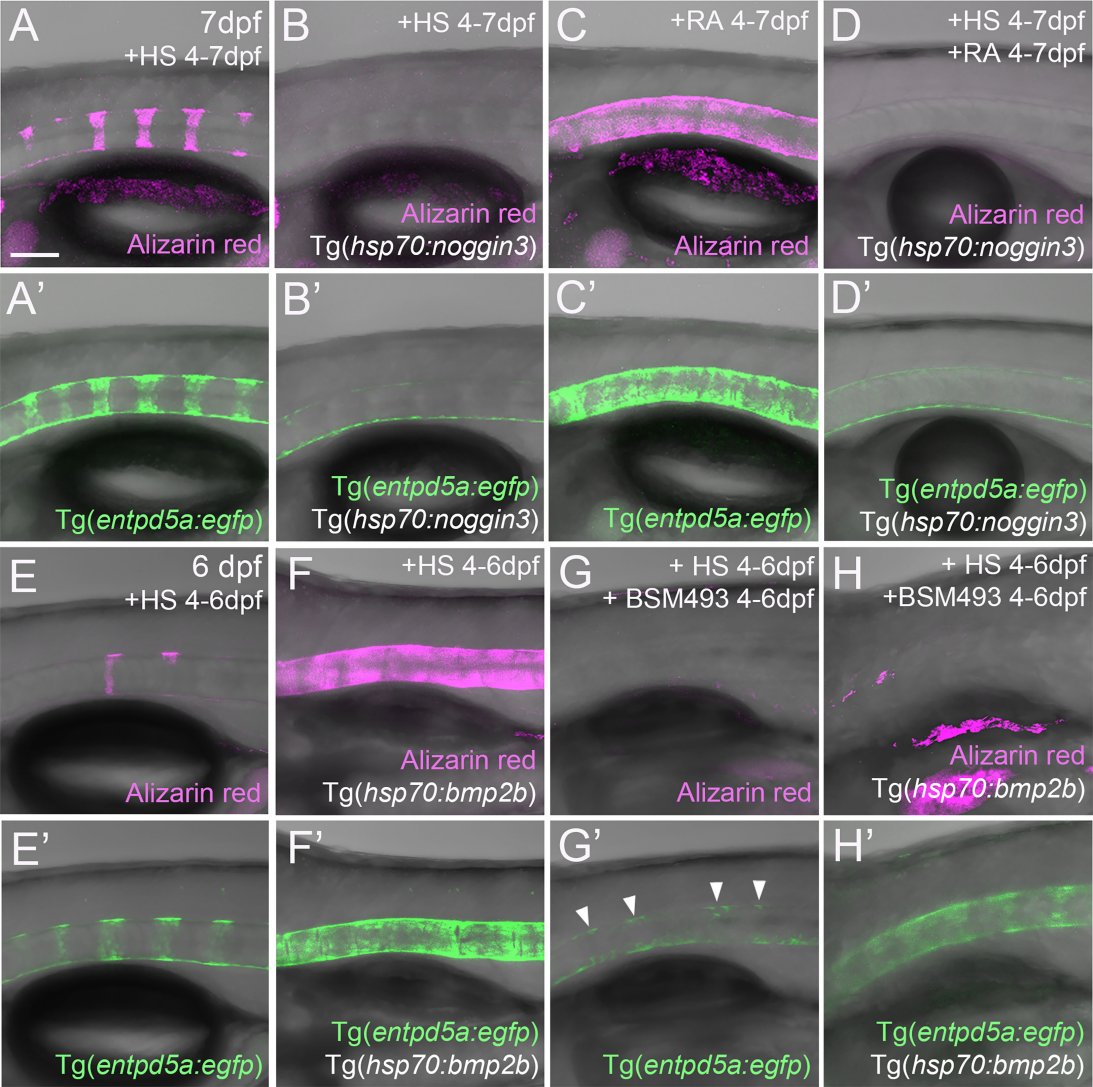
RA cannot promote notochord sheath mineralization when BMP signaling is blocked during initiation phases of mineralization. **(A–D’)** RA fails to induce hyper-mineralization of the notochord sheath while BMP signaling is blocked. **(E–H’)** BMP induced ectopic *entpd5a* activation cannot be hindered by interfering with the intra-cellular signal transduction of the RA pathway. **(A–H’)** Lateral views of transgenic zebrafish larvae shown in merged transmitted and fluorescent light channels with tissue stainings, genotypes and treatments as indicated at an age of 7dpf **(A–D’)** or 6dpf **(E–H’)**. Heat-shocks were performed once a day for 45 minutes at 40°C. Arrowheads in **(G’)** point to faintly EGFP-positive chordoblasts in areas of presumably segmented Tg(*entpd5a:egfp*) expression. Image-pairs X/X’ show identical specimens. Scale bar (shown in **A**, applying to all panels): 100 µm.

Given that both BMP and RA contribute to segmented *entpd5a* expression and notochord sheath mineralization, we wondered whether one acts *via* the other. To test this, we combined gain of function of one factor with loss of function of the other to perform epistasis analyses. To combine loss of BMP with gain of RA signaling, we compared Tg(*entpd5a:egfp*) activity and notochord sheath mineralization (via Alizarin red staining) in Tg(*hsp70:noggin3*) larvae with Tg(*hsp70:noggin3*)-negative siblings, both of which were heat-shocked and additionally treated with RA from 4-7 dpf or, as negative control, with DMSO. As in the studies shown in **Figure 1**, control-treated wild-type siblings showed the expected EGFP reporter expression in the rostral third of the notochord with up to eight mineralized chordacentra formed at 7 dpf (N = 58/58; **Figures 4A, A’**), whereas control-treated heat-shocked *hsp70:noggin3* transgenics lacked both markers, indicating successful blockage of the BMP pathway (N=74/74; **Figures 4B, B’**). Conversely, RA-treated wild-type larvae developed the expected hyper-mineralization/centra fusion phenotype (N=47/47, **Figures 4C, C’**), whereas RA-treated and heat-shocked *hsp70:noggin3* transgenics failed to show any RA-induced responses and displayed the pure BMP loss-of-function phenotype (N=118/118, **Figures 4D, D’**).

In a reverse experiment, combining gain of BMP with loss of RA signaling, we compared Tg(*entpd5a:egfp*) activity and notochord sheath mineralization (via Alizarin red stains) in Tg(*hsp70:bmp2b*) larvae with Tg(*hsp70:bmp2b*)-negative siblings, both of which were heat-shocked and treated from 4-6 dpf with the pharmacological pan-RA receptor inhibitor BSM493 (38) or, as negative control, with DMSO. As in the studies shown in **Figure 1**, control-treated heat-shocked *hsp70:bmp2b* transgenics displayed continuous rather than metameric Tg(*entpd5a:egfp*) expression and Alizarin red incorporation along the notochord (N=21/21, **Figures 4E, E’,** **F, F’**). Comparable uniform Tg(*entpd5a:egfp*) expression was also obtained upon concomitant BSM493-treatment (N=24/24; **Figure 4H’**), however, at considerably lower levels, consistent with the compromised metameric transgene expression in BSM493-treatment of wild-type siblings (N=20/20, **Figure 4G’**). Noteworthy, this moderate ectopic *entpd5a* activation was hardly able to induce notochord sheath mineralization (**Figure 4H**). In sum, RA cannot compensate for the loss of BMP in initiating *entpd5a* expression and consequently notochord sheath mineralization, while BMP cannot compensate for the loss of RA in reaching maximal *entpd5a* expression and thus sheath mineralization levels. In genetic terms, this categorizes BMP as being epistatic to RA in initiating *entpd5a* expression in chordoblasts for notochord sheath mineralization, and RA as being epistatic to BMP in potentiating Entpd5a production.

### BMP and RA signaling do not act *via* each other

**Figure 5:**
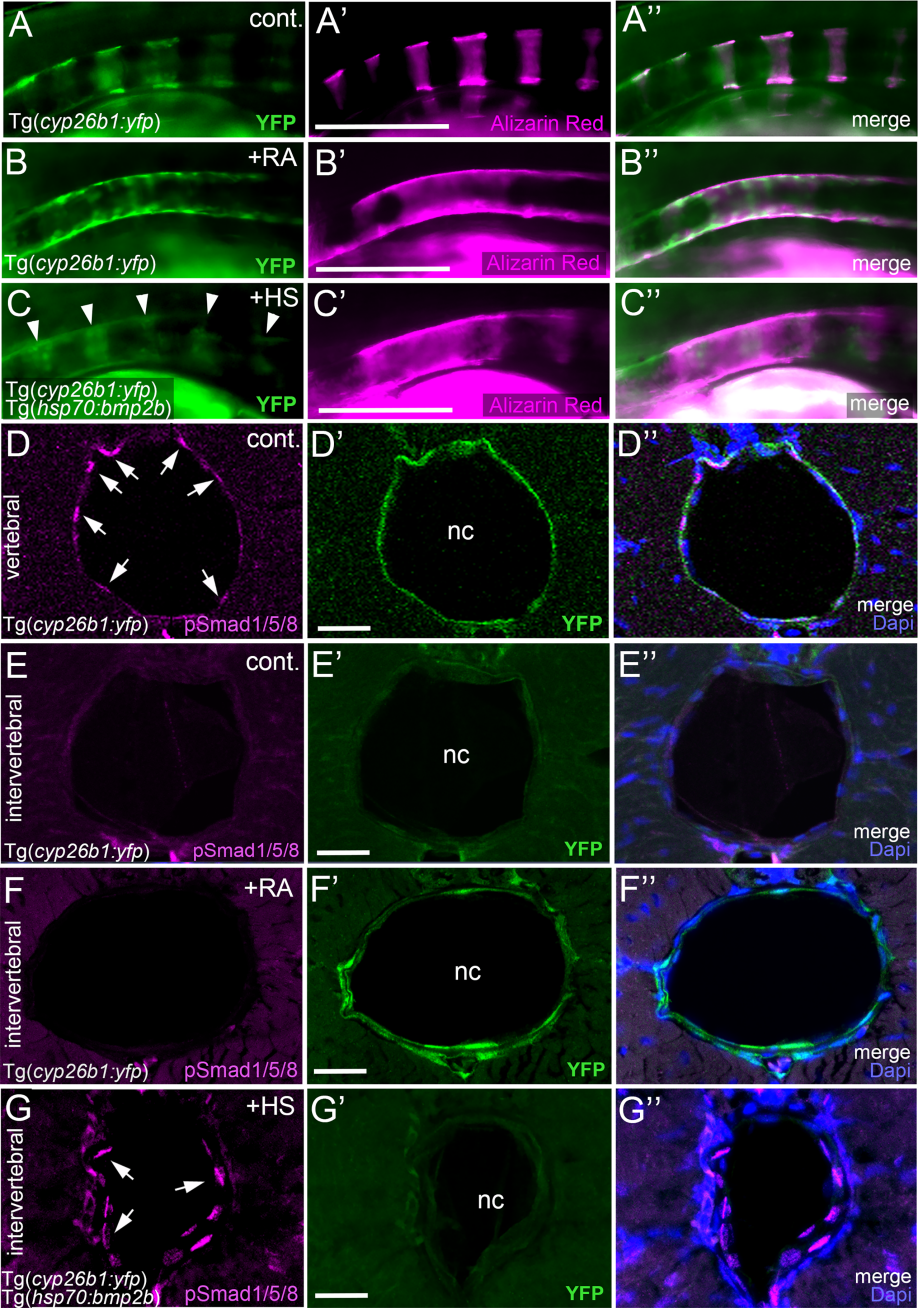
BMP and RA signaling do not act *via* each other. **(A–C’’)** BMP-induced hyper-mineralization is not accompanied by an ectopic activation of the RA signaling reporter Tg(*cyp26b1:yfp*) in the notochord epithelium. Lateral views of notochords immediately dorsal to the swim bladder, anterior to the left. Images were taken by conventional fluorescent light microscopy and are shown as separate 488nm **(A–C)**, 555nm **(A’–C’)** channels and with both channels merged **(A’’–C’’)**. The *cyp26b1:yfp* transgene **(A–C, A’’–C’**’, green**)** labels chordoblasts and serves as a readout for RA-responsiveness. Alizarin red stains the mineralized notochord sheath **(A’–C’**, **A’’–C’’** magenta**)**. Note the remaining segmented YFP signal after global overexpression of BMP2b (**C**, arrowheads). **(D–G’’)** Exposure of larvae to 1 µM RA does not cause ectopic pSmad1/5/8 appearance at intervertebral positions of the chordoblast layer. Cross-sections through the abdominal notochord of 7 dpf control Tg(*cyp26b1:yfp*) **(D–E’’)**, RA-treated Tg(*cyp26b1:yfp*) **(F–F’’)**, and heat-shocked Tg(*cyp26b1:yfp*), Tg(*hsp70:bmp2b*) **(G–G’’)** larvae co-stained *via* immunofluorescence labeling for the activated/phosphorylated BMP signal transducer pSmad1/5/8 and for YFP, encoded by the RA responder transgene. In untreated controls, pSmad1/5/8 reactivity is restricted to vertebral segments co-expressing the RA reporter transgene **(D–D’’)** but absent from intervertebral spaces lacking Tg(*cyp26b1:yfp*) expression **(E–E’’)**. Upon RA-treatment, such intervertebral domains (identified as such by the lack of pSmad1/5/8 activity) display ectopic Tg(*cyp26b1:yfp*) activity, although RA fails to induce ectopic pSmad1/5/8 reactivity **(F-F’’)**. In contrast, as a positive control, ectopic pSmad1/5/8 reactivity in intervertebral regions, identified as such by the lack of Tg(*cyp26b1:yfp*) activity, is obtained by heat shock-induced ubiquitous Tg(*hsp70:bmp2b*) expression **(G-G’’)**. Images represent single confocal slices shown as separate channels 555nm in magenta **(D–G)**, 488nm in green **(D’–G’)** and merged with Dapi **(D’’–G’’)**. Arrows in **(D, G)** point at exemplary pSmad1/5/8 positive chordoblasts. Whole figure: Genotype, age, labeling and treatments as indicated. Heat-shocks were performed once a day for 45 minutes at 40°C from 4-7 dpf. RA was applied constantly from 4-7 dpf. Scale bars: 200 µm **(B)**; 20 µm **(D’–G’)**. cont., vehicle-treated control; nc, notochord; HS, heat shock; RA, Retinoic acid.

The described epistatic relationships between BMP and RA could mean that RA acts upstream of BMP, with RA inducing BMP signaling to initiate notochord sheath mineralization, but also that BMP acts upstream of RA, with BMP inducing RA signaling to potentiate *entpd5a* expression. This would imply a switch in the linear order of action of the two during the course of notochord sheath mineralization, with RA first acting upstream and later downstream of BMPs. This seems quite unlikely. To look into these options more directly, we studied whether gain of BMP signaling induces gain of RA signaling and/or vice versa. To address RA signaling, we determined the expression of the *cyp26b1:yfp* transgene, which has been used as an *in vivo* reporter for RA signal reception by zebrafish chordoblasts before (24). As previously reported, untreated Tg(*cyp26b1:yfp*) controls displayed iterative segments of the notochord epithelium labeled by YFP, overlapping with the sites of chordacentra mineralization visualized by alizarin red staining (N=12/12; **Figures 5A, A’, A’’**). Exposure of Tg(*cyp26b1:yfp*) transgenics to RA led to the expected expansion of *yfp* expression in the chordoblast layer that was accompanied by a hyper-mineralization of the notochord sheath (N=33, **Figures 5B, B’, B’’**). However, global Bmp2b overexpression in 4-7 dpf heat-shocked *hsp70:bmp2b* transgenics did not alter the segmented appearance of YFP-positive chordoblast clusters (N=54, **Figure 5C**), although it led to a similar notochord sheath hyper-mineralization (**Figure 5C’**) as the YFP-expanding RA treatment (**Figure 5B’**). Thus, BMP does not drive notochord sheath mineralization by inducing or promoting RA signaling.

The reverse, however, also does not seem to be the case: in co-labeled untreated controls, transverse sections through vertebral regions display chordoblasts that are positive for both pSmad1/5/8 and Tg(*cyp26b1:yfp*) activity (N=3; **Figures 5D–D’’**), whereas chordoblasts in intervertebral regions lack both pSmad1/5/8 and Tg(*cyp26b1:yfp*) activity (N=3; **Figures 5E–E’’**). Consistent with the data described above (**Figures 5C–C’’**), upon forced ubiquitous BMP expression in Tg(*hsp70:bmp2b*) fish heat-shocked from 4-7 dpf, such intervertebral regions remained Tg(*cyp26b1:yfp*) negative, although displaying ectopic pSmad1/5/8 reactivity (N=5, **Figures 5G–G’’**). In contrast, upon RA treatment, intervertebral regions have become positive for Tg(*cyp26b1:yfp*) activity while still lacking pSmad1/5/8 reactivity (N=7, **Figures 5F–F’’**). Therefore, although BMP seems epistatic to RA in initiating *entpd5a* expression and notochord sheath mineralization (**Figures 4A–D**), RA does not seem to act *via* BMPs.

### RA can promote notochord sheath mineralization independently of BMP signaling at later stages of vertebral column formation

**Figure 6:**
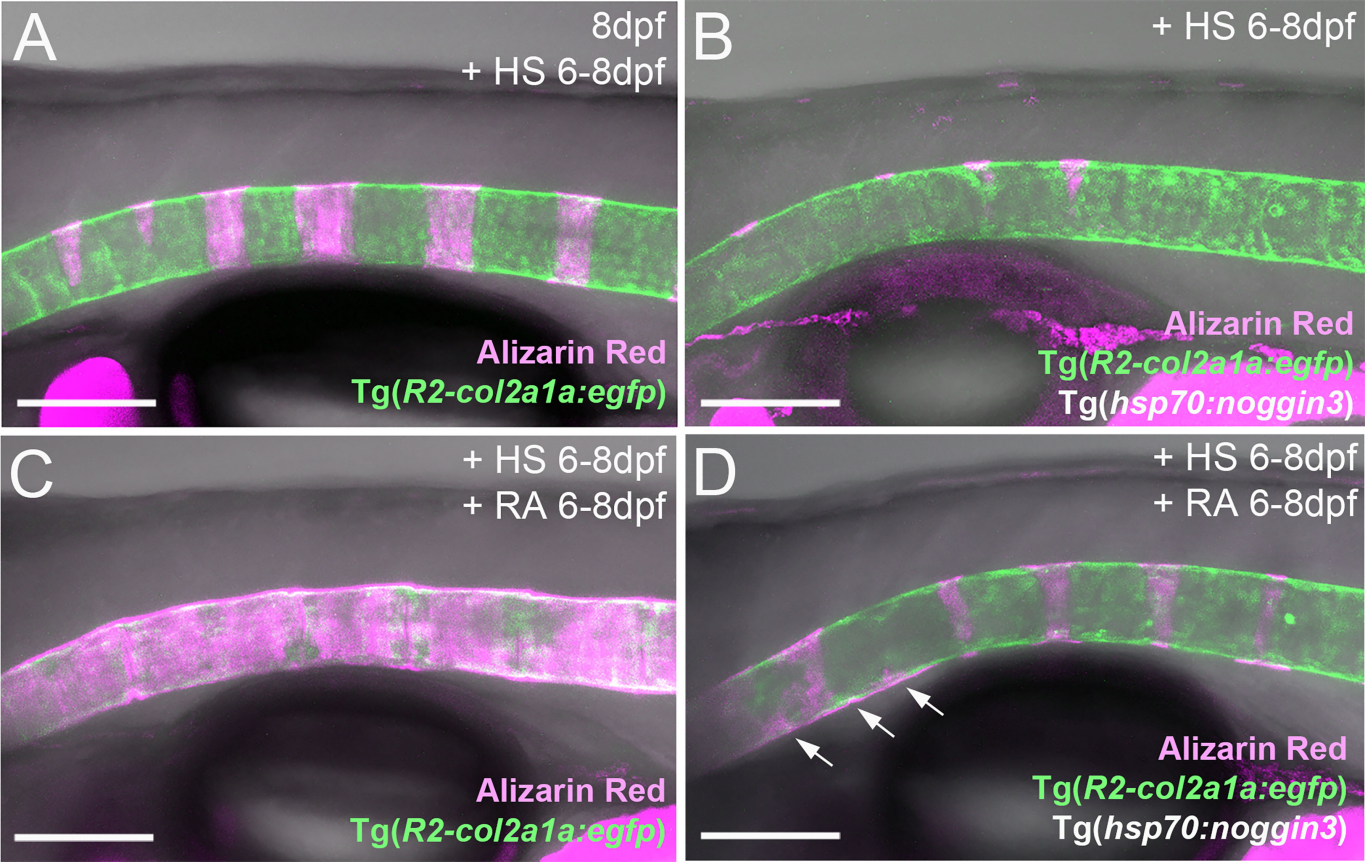
RA can promote notochord sheath mineralization independently of BMP signaling at later stages of vertebral column formation. **(A–D)** Blockage of BMP signaling with synchronous activation of the RA pathway from six to eight dpf results in larvae with less severe disrupted notochord sheath mineralization while chordoblasts show clear marks of RA responsiveness (decreased Tg(*R2-col2a1a:egfp*) activity and less distinct cellular morphology). Lateral views of transgenic zebrafish larvae shown in merged transmitted and fluorescent light channels with tissue staining, genotypes and treatments as indicated at an age of 8dpf **(A–D)**. Heat-shocks were performed once a day for 45 minutes at 40°C. Arrows in **(D)** point at zones of ectopic mineralization. Scale bars: 150 µm.

An action of RA upstream of BMP to initiate *entpd5a* expression for notochord sheath mineralization also seems unlikely in the light of our formerly published data, according to which zebrafish larvae can respond to BMP overexpression by ectopic notochord sheath mineralization approximately two days earlier than to RA treatment (19). Thus, the epistatic relationship between RA and BMP revealed in the studies with combined loss of BMP and gain of RA activity described above (**Figures 4A–D**), might need to be interpreted differently. An alternative explanation could indeed be consecutive, temporally separated functions of the two, with RA only being able to promote chordoblasts and their mineralizing activity AFTER they have received BMP signals inducing a crucial earlier step of chordoblast differentiation. To test this notion, we performed the epistasis analyses described above (**Figures 4A–D**) at a later time window of vertebral development, co-treating larvae from 6-8 dpf, rather than from 4-7 dpf. Global overexpression of *noggin3* during that later time window arrested the mineralization process in a 6 dpf like-state, with beginning and subtle mineralizations of the first four to five centra (N=35, **Figure 6B**), compared to the stronger and posteriorly advanced mineralization of the first ten centra in non-transgenic controls (N=25, **Figure 6A**). Heat-shocked non-transgenic specimens hyper-mineralized their notochord sheath when exposed to RA from 6-8 dpf (N=33, **Figure 6C**). Remarkably and different to the corresponding experiment performed from 4-7 dpf (**Figure 4D**), upon combined Noggin3 and RA treatment from 6-8 dpf, the loss-of-BMP-function phenotype did not completely prevail over the gain-of-RA-function effect. Instead, we detected an intermediate phenotype with increased, but not fully normalized mineralization levels in the first seven centra and some signs of ectopic mineralization at the ventral side of the notochord (N=28, **Figure 6D**), indicating that later loss of BMP cannot fully abrogate RA-induced mineralization. In addition, compared to untreated siblings (**Figure 6A**), Tg(*R2-col2a1a:egfp*) expression in chordoblasts of double-treated larvae was diminished (**Figure 6D**), indicating that even in the absence of such late BMP signaling, RA can induce the progression of chordoblasts from matrix-producing to matrix-mineralizing cells.

Together with their differential effects on *col2a1* and *entpd5a* expression described above (**Figures 1**, **2**), these data point to consecutive functions of BMP and RA during subsequent steps of chordoblast differentiation and activity. Hereby, BMP promotes the progression of purely matrix-producing to transitory matrix-producing and simultaneously matrix-mineralizing cells, while RA can only affect the latter (and later occurring) stage *via* further promoting its matrix-mineralizing capabilities while attenuating its matrix-producing activity. These findings are consistent with the former discovery of the corresponding three chordoblast subpopulations *via* FACS sorting of transgene-labeled chordoblasts and comparative transcriptome analysis (25). Along these lines, the incomplete normalization of the typical 8 dpf pattern of notochord sheath mineralization in larvae double-treated with Noggin3 and RA from 6-8 dpf would be due to the fact that at 6 dpf, not all usually mineralization-promoting chordoblasts along the notochord and within presumptive centra segments have received the pre-determining BMP signal as yet.

## Discussion

It is certainly not surprising that a family of highly conserved signaling factors that have initially been identified as regulators of mammalian connective tissue mineralization (1, 2) (as reflected by their name: Bone Morphogenetic Proteins) take action in this particular function also in teleosts. Accordingly, BMP signaling has for instance been shown to have an impact on bone formation during zebrafish facial skeleton development (46) and fin regeneration (47). Also, as reported by us earlier, global BMP overexpression between 2 and 4 dpf leads to a fusion of vertebral centra in zebrafish larvae (19). In this follow-up study, we set out to shed further light on the endogenous function of BMP and its relationship to RA signaling in this developmental context.

### Chordoblasts, the primary skeletogenic cells of the developing teleostean vertebral centra anlagen, are direct cellular targets of consecutive positive regulation by BMPs and RA

**Figure 7:**
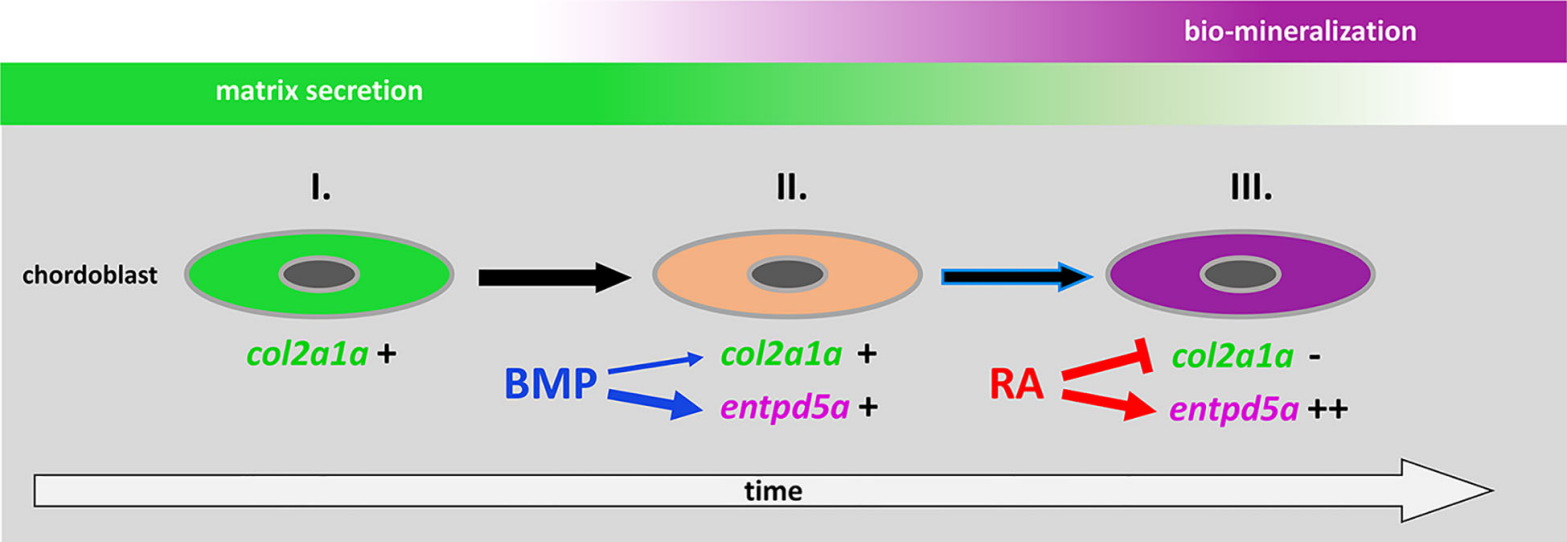
Proposed model for the consecutive roles of BMP and RA to induce the stepwise progression of chordoblasts from solely matrix-producing towards solely matrix-mineralizing cells. I. Chordoblast contribution to chordacentra formation starts with collagen II-based matrix (notochord sheath) production. II. Next, cells progress into a matrix-secreting and -mineralizing transitory stage induced by BMP, with an induction of *entpd5a* expression, required for matrix mineralization (thick blue arrow) and with some minor positive effect on *col2a1a* expression (thin blue arrow). III. Lastly, RA signaling enhances and maintains *entpd5a* transcription (thick red arrow), thereby reaching Entpd5a levels that allow for sustained bio-mineralization of the notochord sheath. At the same time RA represses *col2a1a* transcription to attenuate matrix secretion. Chordoblasts can only enter step III after they have experienced BMP signaling beforehand (black arrow with blue lining).

It was an unexpected and remarkable finding of recent years that the initial mineralization events in early teleost vertebral column development are not driven by sclerotome-derived osteoblasts, but by cells from the notochord, the chordoblasts (23–25). Noteworthy, the cellular and biochemical mechanisms underlying the formation of such chordacentra by chordoblasts are very similar to what we know from cells of the regular osteogenic lineage: secretion of a special extracellular matrix (ECM) followed by its bio-mineralization. In typical bone formation, osteoblasts secrete collagens and proteoglycans to generate a specialized ECM called osteoid as a first step. Subsequently, osteoblasts differentiate further to preosteocytes, which stop matrix production and instead initiate its calcification, thereby finalizing the process of ossification (32, 33). Chordoblasts do basically the same (**Figure 7**): first, they secrete a type II collagen-based extracellular matrix, the notochord sheath; thereafter, they express *entpd5a* to allow for bio-mineralization of this sheath. Analogously to preosteocytes, the fully mineralizing chordoblasts gradually tune down matrix production (24, 25). Of note, the functional progression of chordoblasts from merely matrix-secreting towards merely mineralizing cells runs through a transitory phase. The latter is characterized by an onset of the Entpd5a-mediated mineralization process while notochord sheath production still pursues (25).

Here, we show that BMP – like RA - is capable of expanding *entpd5a* transcriptional activity in chordoblasts with the consequence of increased notochord sheath mineralization. But in contrast to RA, the BMP-induced effect is obviously not directly paralleled by a downregulation of matrix production. In fact, our results even point to a moderately positive impact of forced BMP signaling on *col2a1a* expression, contrasting the negative effect of increased RA signaling (**Figure 2A**), while the thickness of the notochord, again in contrast to the negative effect of RA signaling, is not significantly altered (**Figure 2B**). Thus, BMP’s function is obviously not to silence chordoblasts’ matrix secretion for the sake of mineralization, but to direct chordoblasts into that transitory phase of synchronized ECM secretion and mineralization – comparable to an early preosteocyte (32, 33). In contrast, RA is required for the next and subsequent step, inducing the progression of chordoblasts into a later stage characterized by aborted matrix production and elevated mineralizing activity (24), reflected by *col2a1a* downregulation, reduced thickness of the notochord sheath and several-fold *entpd5a* upregulation in RA-treated larvae (**Figures 2A, B**, **7**). Accordingly, given the BMP-driven mineralization during the transitory phase, loss of RA signaling does not lead to a complete loss, but just severely reduced levels of notochord-intrinsic *entpd5a* activity (**Figures 4E’, G’**), which seem higher than in the case of loss of BMP signaling (**Figures 4A’, B’**). Such a consecutive function of BMP and RA also explains our former findings that zebrafish larvae can respond to BMP overexpression by ectopic notochord-sheath mineralization approximately two days earlier than to RA treatment (19). Furthermore, the epistasis analysis presented here indicates that chordoblasts need to run through this BMP-dependent transitory stage before becoming responsive to RA (**Figure 7**). Thus, RA treatment fails to induce an up-regulation of *entpd5a* expression in chordoblasts when they are co-exposed to high levels of the BMP inhibitor Noggin3 during early phases of vertebral column formation, when chordacentra mineralization is initiated (4 dpf; **Figures 4A–D’**), whereas approximately two days later, when at least some of the chordoblasts have received the BMP signal and have entered the transitory stage, RA can stimulate chordacentra mineralization in the absence of BMP signaling (**Figure 6**). Future single cell transcriptome analyses of chordoblasts under these different conditions will be necessary to reveal by which means the preceding BMP signaling prepares ground for the later RA signal. Apparently, it is not achieved by inducing RA signaling, for instance by activating the expression of RA-synthesizing Aldehyde Dehydrogenase enzymes, or by making chordoblasts competent for the reception of RA signals, for instance by inducing the production of RA receptors. Thus, BMP overexpression does not lead to enhanced RA signaling in chordoblasts, as indicated by the unaltered expression of the reporter transgene *cyp26b1:yfp* that labels RA-receiving cells (**Figures 5A–C’’**).

### BMPs, rather than RA, might generate the metameric pattern in the initially unsegmented chordoblast layer of the notochord

A puzzling and still unresolved question is how the pattern of iterative *entpd5a* expressing segments within the otherwise uniform appearing chordoblast layer gets implemented in the first place. Different working-models have been proposed, ranging from a notochord-inherent molecular pattern generator up to an induction by external signals that are secreted by primarily segmented sources (22–26). To sort things out it is of course crucial to identify the involved molecular regulators, and RA as well as components of the Notch signaling system have already been implicated in this context (24, 25). Here, we provide evidence that BMP signaling also feeds into the molecular mechanisms that construct the early vertebral column anlage in zebrafish. Based on our data, BMP appears to direct chordoblasts from the merely ECM-producing into a transitory stage, thereby placing it functionally upstream of the RA-dependent manifestation of the merely mineralizing phase. Similarly, Notch signaling has been found to have impact on the initial transition from matrix-secreting to *entpd5a*-expressing chordoblasts (25). Further research is needed to dissect the functional and genetic correlation between BMP and Notch. In other contexts, ranging from vein formation in Drosophila wings (48) to zebrafish heart regeneration (49), Notch signaling has been reported to act upstream of BMP/Dpp signaling. However, as in the case of BMP and RA described here, corresponding epistasis analyses have to be taken with caution, as one factor being epistatic to another does not necessarily mean that it acts downstream, but could rather be involved in an earlier step of the biological process. Thus, temporally controlled double treatments at different time points are necessary to allow definitive answers.

In fact, based on its time of action, its functions and its formerly demonstrated diffusivity (50, 51), BMP itself might be the initial pattern generator during zebrafish vertebral column segmentation. Future research will aim to identify the specific BMP ligands and their cellular sources involved in these early steps of chordoblast activation and notochord epithelial segmentation. Analyses of secreted BMP inhibitors like Noggin, Chordin or Sclerostin (52) and their spatial distributions will further elucidate potential mechanisms to restrict the growth of individual centra along the anteroposterior axis of the notochord and to avoid centra fusions. Such inhibitors could for instance be expressed in a pattern complementary to that of the mineralization-inducing BMPs, thus in, or adjacent to, presumptive intervertebral regions (53). Alternatively, they could be co-expressed with the BMPs in, or adjacent to, presumptive vertebral regions, constituting a dynamic reaction-diffusion mechanism within the chordoblast layer, similarly to how it has been formerly demonstrated for other activator-inhibitor systems during zebrafish embryogenesis (54, 55).

### Similarities and differences in the roles of BMP and RA signaling during vertebral column formation in zebrafish versus mouse and human

Two prominent human syndromes display vertebral fusions caused by altered BMP signaling, which in both cases are restricted to cervical vertebrae of the neck: Klippel-Feil-Syndrome (KFS) caused by loss of GDF6 or GDF3, both BMP family members (5–7), thus contrasting our data that gain of BMP signaling causes chordacentra fusions in zebrafish, and Fibrodysplasia ossificans progressiva (FOP), caused by gain of BMP signaling *via* a mutated, constitutively active version of the BMP type I receptor ACVR1/ALK2 (9–11), consistent with our zebrafish data.

We can only speculate about the apparently opposite roles and effects of BMP signaling in human KFS versus human FOP and zebrafish chordacentra fusion. It has been reported that in difference to the thoracic and abdominal vertebrae, skeletogenic cells forming the KFS-affected cervical vertebrae derive from the neural crest rather than the somitic sclerotome (56). Thus, and in light of the much earlier manifestation of the vertebral fusion phenotype in KFS compared to FOP, resulting from early segmentation defects (see Introduction), it is tempting to speculate that in amniotes, GDF3/6 might play a specific early role in the development of such neural crest precursors that is not relevant in teleosts, where neural crest cells do not contribute to vertebra formation. Thus, BMP-induced chordacentra fusions in zebrafish, although similarly to KFS occurring at the level of the vertebral bodies/centra themselves, are definitively no model for human Klippel-Feil syndrome.

FOP is characterized by orthotopic and heterotopic ossifications at multiple sites, among them fusions at the level of lateral masses and neural arches of cervical vertebrae (ossification of the vertebral facet joints) (9–12), thus crucially contrasting the fusion type in KFS and in our zebrafish models described here, which are characterized by fusions at the level of the vertebral bodies/centra themselves. These orthotopic and heterotopic endochondral ossifications of FOP have been attributed to ALK2-mediated excessive chondrogenic differentiation of mesenchymal progenitor cells recruited to inflamed “flare-up” sites (57). Given that endogenous RA signaling has been known to be normally attenuated during chondrogenesis, that this attenuation is required for chondrogenic differentiation (58, 59) and that exogenous RA agonists can block chondrogenesis (60), it seemed feasible to prevent or alleviate such ossifications of FOP by blocking early ectopic chondrogenic differentiation processes *via* a pharmacological enhancement of RA signaling. And indeed, results obtained with the RA receptor γ (RARγ) agonist Palovarotene were so convincing that it has recently been approved for FOP therapy in human (61–63). Strikingly, the strong alleviation of heterotopic ossification defects in Palovarotene-treated genetic FOP mice was accompanied by significant reductions in pSmad1/5 levels (61, 63), suggesting that at the affected sites, RA normally acts upstream and as a negative regulator of BMP signaling. This is in striking contrast to our findings that during early mineralization steps of the developing vertebral column in zebrafish, BMP and RA have similar, rather than opposite effects, that BMP acts earlier, rather than as a mediator of RA, and that BMP and RA signaling do not affect each other. Furthermore, vertebral fusions in FOP are restricted to the neural arches (leaving the vertebral bodies unaffected) and to the cervical vertebrae, whereas in zebrafish, overexpression of BMP leads to fusions of the centra/vertebral bodies themselves and occurs along the entire length of the developing vertebral column.

In light of these differences, in addition to the long-known morphological differences between developing vertebral columns of amniotes and teleosts, as well as the strikingly different cellular contributions to vertebra formation in amniotes (neural crest- and sclerotome-derived chondrocytes and osteoblasts) versus teleosts (notochord-derived chordoblasts and sclerotome-derived osteoblasts) (28, 30), the mechanisms of early backbone formation and segmentation might appear so fundamentally different between the two clades that data obtained in zebrafish could be considered completely irrelevant for a better understanding of vertebral malformations in human.

On the other hand, it cannot be ruled out that the antagonistic correlation between RA and BMP signaling revealed in the mouse FOP models primarily applies to other sites of heterotopic ossifications, but not to the orthotopic ossifications in cervical vertebrae. Indeed, there is evidence that here, BMP and RA might act as partners, rather than opponents, similar to their cooperation during vertebral column development in zebrafish demonstrated in this work. Thus, in mouse, gain of endogenous RA signaling upon pharmacological inhibition of Cyp26 RA inhibitors leads to fusions of neural arches of cervical vertebrae (19) similar to those obtained by gain of endogenous BMP signaling upon genetic loss of the BMP inhibitor Noggin in mouse and FOP in human (12, 20). This suggests that at least some crucial, not well understood aspects of vertebral column segmentation and vertebrae fusion ARE mechanistically conserved among zebrafish, mouse and, possibly, human. More and more direct comparative studies of BMP and RA signaling during vertebral column formation in teleosts and mammals, including detailed expression pattern analyses of BMPs, BMP inhibitors, RA-synthesizing and RA-metabolizing Cyp26 enzymes, as well as cell type-specific loss-of-function experiments, will be necessary to unravel such conserved aspects within otherwise rather clade-specific and differential developmental programs (28).

Furthermore, future studies need to address whether for zebrafish, the concept of consecutive functions of BMP and RA for a stepwise transition of matrix-producing to matrix-mineralizing skeletogenic cells also applies to mineralizing tissues outside the notochord. Increased and precautious mineralization induced by gain of RA or loss of its inhibitor Cy26b1 have been reported for multiple other zebrafish bones, formed either *via* perichordal or intramembranous ossification, such as the ceratohyale and the opercle of the craniofacial skeleton (19), the fin rays (64) and the calvarial plates of the skull (34, 65). Of note, for the mouse skull, application of the BMP inhibitor Noggin has been shown to rescue RA-induced craniosynostosis (66), pointing to mineralizing-promoting effects of both RA and BMP, contrasting their opposite effects during FOP and consistent with our data revealed here for the developing zebrafish vertebral column, but with a function of BMP downstream of RA that is opposite to the order revealed here for the zebrafish vertebral column.

## Data availability statement

The original contributions presented in the study are included in the article/**Supplementary Material**. Further inquiries can be directed to the corresponding author.

## Ethics statement

The animal study was reviewed and approved by LANUV Nordrhein-Westfalen.

## Author contributions

H-MP and MH contributed to the conception and design of the study. IR-Q and H-MP performed the experiments, analyzed samples and collected data. H-MP conducted the statistical analysis. H-MP and MH wrote the manuscript. All authors contributed to the article and approved the submitted version.

## Supplementary material

Supplementary Figure 1 Ablation of chordoblasts neither disrupts tissues outside the notochord nor does it impair overall integrity of the larvae. **(A, B)** Lateral view with anterior to the left of a wild-type control **(A)** and a Tg(*R2-col2a1a:CFP-NTR*) transgenic larva **(B)** both of them Mtz treated as indicated, at 7 dpf. Even though the chordoblast-ablated animal is a bit shorter than the control, it looks overall intact, has an inflated swim bladder and displays no signs of increased necrosis in tissues outside the notochord. **(A’, B’)** depict corresponding magnifications of notochords from the specimens shown in **(A, B)**. As in the control larva **(A’)**, vacuolated chordocytes persist after chordoblast ablation upon Mtz application from 4dpf to 6.5dpf **(B’)**. **(C, D)** Semi-thin transverse sections through posterior abdominal regions of Mtz-treated wild-type and Tg(*R2-col2a1a:CFP-NTR*) larvae. Except for the collapse of the notochord that is caused by the chordoblast ablation in combination with the applied histology procedures, all other tissues of Mtz-treated *R2-col2a1a:CFP-NTR* transgenic larvae appear unaffected **(D)**. Scale bars: 200 µm **(A, B)**, 100 µm (A’,B’), 20 µm **(C, D)**. Abbreviations: nc = notochord, ns = notochord sheath.
