# Supplementary figures and images for: Direct BMP signaling to chordoblasts is required for the initiation of segmented notochord sheath mineralization in zebrafish vertebral column development
Source: Front Endocrinol (Lausanne). 2023 May 8;14:1107339. doi: 10.3389/fendo.2023.1107339 (PMC10200950; doi:10.3389/fendo.2023.1107339)

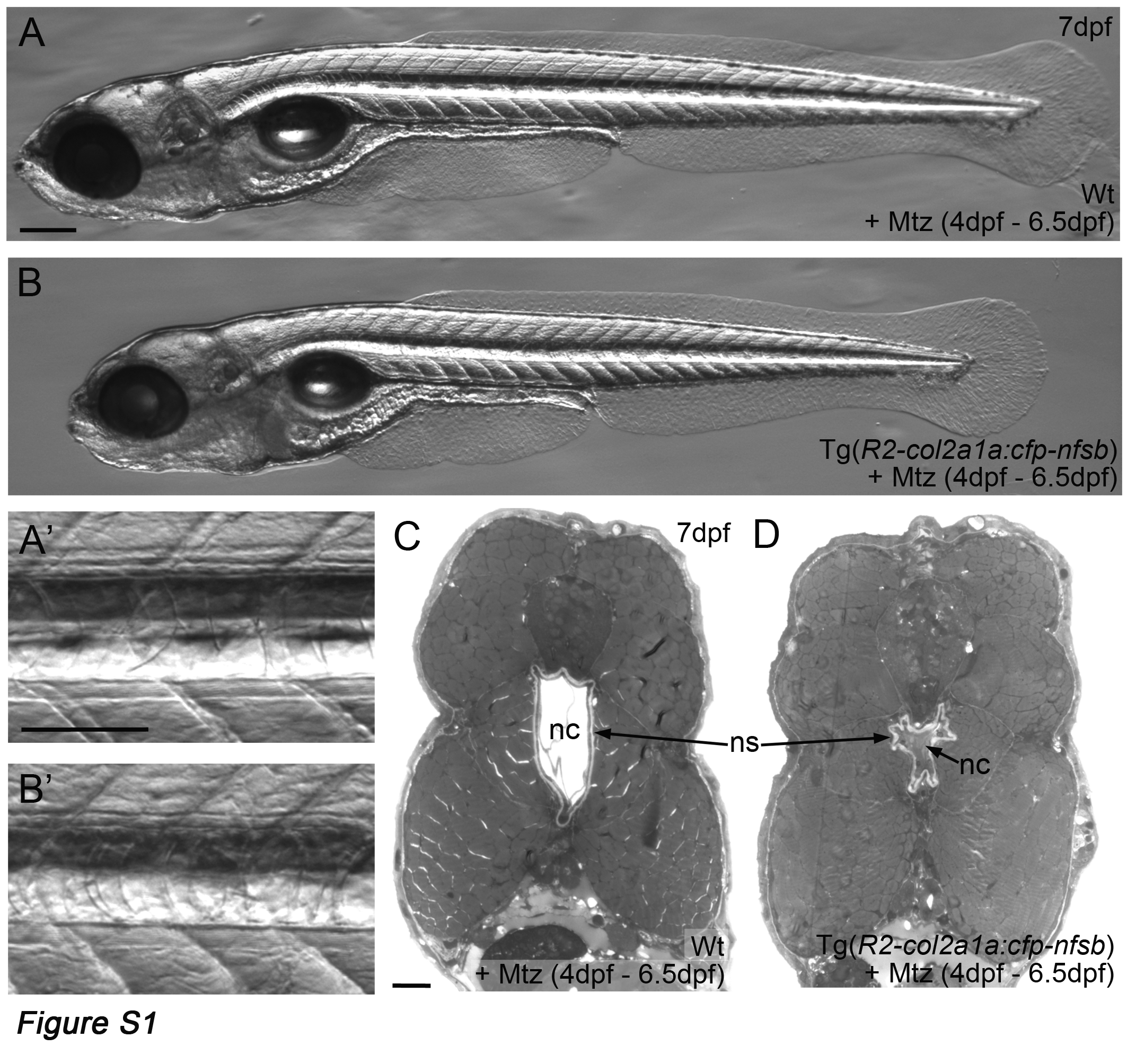

Supplement: Supplementary Figure 1 — Ablation of chordoblasts neither disrupts tissues outside the notochord nor does it impair overall integrity of the larvae. (A, B) Lateral view with anterior to the left of a wild-type control (A) and a Tg(R2-col2a1a:CFP-NTR) transgenic larva (B) both of them Mtz treated as indicated, at 7 dpf. Even though the chordoblast-ablated animal is a bit shorter than the control, it looks overall intact, has an inflated swim bladder and displays no signs of increased necrosis in tissues outside the notochord. (A’, B’) depict corresponding magnifications of notochords from the specimens shown in (A, B). As in the control larva (A’), vacuolated chordocytes persist after chordoblast ablation upon Mtz application from 4dpf to 6.5dpf (B’). (C, D) Semi-thin transverse sections through posterior abdominal regions of Mtz-treated wild-type and Tg(R2-col2a1a:CFP-NTR) larvae. Except for the collapse of the notochord that is caused by the chordoblast ablation in combination with the applied histology procedures, all other tissues of Mtz-treated R2-col2a1a:CFP-NTR transgenic larvae appear unaffected (D). Scale bars: 200 µm (A, B), 100 µm (A’,B’), 20 µm (C, D). Abbreviations: nc = notochord, ns = notochord sheath. [file Image_1.tif]
